# Dependability of Osstell ISQ's for measuring implant stability

**DOI:** 10.6026/973206300200921

**Published:** 2024-08-31

**Authors:** Vijay Parmar, Nureldeen AN Elhammali, Omar Basheer Altaher Mohammed, Meghna Chauhan, Pushkar Gupta, Abhigyan Manas, Ankita Raj, Hrithik Chetani

**Affiliations:** 1 Department of Prosthodontics, Goenka Research Institute of Dental Science, Gandhinagar, Gujarat, India; 2 Department of Oral and Maxillofacial Surgery, Faculty of Dentistry, Sirte University, Libya; 3 Department of Oral and Maxillofacial Surgery, Sebha Dental Faculty, Sebha University, Libya; 4 Department of Dentistry, World College of Medical Sciences & Research and Hospital, Jhajjar, Haryana, India; 5 Department of Prosthodontics and Crown & Bridge, Hitkarini Dental College and Hospital, Jabalpur, MP, India; 6 Department of Oral and Maxillofacial Surgery, Babu Banarsi Das College of Dental Sciences, Lucknow, UP, India; 7 Department of Oral and Maxillofacial Surgery, Rama Dental College, Hospital and Research Centre, Kanpur, Uttar Pradesh, India; 8 Department of Oral Medicine and Radiology, Kalinga Institute of Dental Sciences, KIIT Deemed to be University, Patia, Bhubaneswar-751024, Odisha, India

**Keywords:** Dental implants, resonance frequency analysis, ISQ, implant stability, Osstell

## Abstract

Determining the optimal loading schedule and measuring implant stability at different times are critical tasks. Numerous tools have been created to assess implant-bone stability as a sign of a well-treated implant. Thus, the objective of this cross-sectional study was to estimate the validity of the Osstell ISQ system for assessing implant stability. Osstell ISQ was used to complete implant stability registers for 60 implants across 18 patients. Two distinct SmartPegs (types I and II) were used to complete six measurements on each implant, or three measurements in a row with each transducer. In the 1st, 2nd, and 3rdmeasurements with SmartPegs I and II, the average ISQ was 71.36, 71.31, and 71.65, and 71.02, 71.58, and 71.76, respectively. For SmartPegs I and II, equivalent values or variations below three ISQ points were found in 46.3% and 58.6% of the cases, respectively. Both SmartPegs had an intraclass correlation coefficient of 0.96, and they also had repeatability and reproducibility of 0.96. An intra-class correlation coefficient analysis reveals nearly excellent repeatability and reproducibility for the RFA system Osstell ISQ. Measurements of Osstell ISQ have excellent repeatability.

## Background:

Dental implants are becoming more and more popular as a tooth replacement option these days. With survival and success rates comparable to those attained with conventional load, immediate load in implants has shown to be a predictable treatment [1].The durability and osseointegration of dental implants are critical components of their success. Implants must osseointegrated with alveolar bone in order to operate and sustain masticatory pressure. Implant osseointegration is the process by which the titanium surface of the implant comes into close contact with the alveolar bone without the need for soft tissue to about it. There are two phases of osseointegration: primary and secondary. During the initial stage, mechanical interaction with cortical bone is the primary means of achieving implant stability. On the other hand, bone remodelling and regeneration are used to achieve implant stability in the secondary stage [2]. Primary implant stability upon implantation is influenced by a number of variables, including implant design, surgical technique, and bone density. Since it restricts micro-movements in the contact between the implant and the bone, primary implant stability is the key determinant of successful initial loading [3].

After implant implantation, initial stability has typically been evaluated based on bone quality and movement using the Lekholm & Zarb jaw quality scale [4]. One can forecast a dental implant's prognosis based on its stability. An implant's stability was defined as its capacity to withstand vertical, rotational, and horizontal stresses. This capacity was used as a proxy for osseointegration and effective healing [5]. Stability refers to an implant's ability to sustain load in the axial, lateral, and rotational directions. The quantity and quality of bone, surgical technique, and implant design all affect primary stability. During the first week following implantation, the primary stability is crucial to the stability of the implant. After that, it dramatically declines to negligible levels at around two weeks. The development of new bone and bone remodelling at the implant-bone contact are prerequisites for secondary stability. A biological mechanism forms the basis of the secondary stability [6, 7].

Implant stability measures (ISQ) are utilised as a predictive sign for potential implant failure and as an indirect indicator to establish the time range for practical implant loading in clinical practice [7]. The degree to which an implant resists deformation in response to an applied force is known as its stiffness. The mechanical characteristics of the bone at the implant placement site and the degree, to which the fixture is engaged with the osseous tissue, as dictated by implant geometry and surgical technique, are the two key factors that affect primary stability [8].Primary stability can be measured in a number of ways, some of which entail non-invasive quantitative analysis like damping capacity analysis (DCA) and resonance frequency analysis (RFA). The Osstell ISQ Mentor (Osstell, Göteborg, Sweden) is one of the RFA devices. It measures resonance frequency values using a smart-peg sensor paired with an implant fixture. The resonance frequency measurements are then translated into an arbitrary implant stability scale value known as the implant stability quotient (ISQ). Implant damping properties are measured by DCA systems according to contact time [5]. The implant stability test (IST) is conducted using percussion with the more current AnyCheck® system [9].

Insertion torque, percussion testing, and radiographic examination are non-invasive techniques for assessing implant stability. There are a few things to take into account even though radiographic examination is non-invasive and offers useful information regarding the state of the implant and surrounding bone. Images from radiography can get distorted [10]. Resonance frequency analysis, often known as the OTTL method, is a non-invasive clinical technique that was described by Meredith *et al.* Sävedalen, Sweden-based Integration Diagnostics Ltd. has been designing Osstell devices since 1999. For implant stability testing, this device has undergone multiple generations in the last ten years [3,11]. A piezoelectric transducer is used to generate a sinusoidal signal at a certain frequency intended to cause the implant to vibrate, the non-invasive diagnostic method works. The instrument measures the implant's resistance to vibration, converting it into an ISQ value (implant stability quotient) on a scale of 0 to 100, where 100 represent the maximum implant stability [3]. RFA has emerged as one of the most popular methods for evaluating implant stability in clinical settings in recent years. The overall mechanical stability of an implant is positively reflected by the ISQ score [7].

Two devices can be used to evaluate stability: the PeriotestTM device, which is based on damping capacity assessment technology and the Osstell® ISQ, device, which is based on resonance frequency analysis technology. Tapping or lightly striking the implant is part of the percussion test procedure. Implant stability can also be objectively measured using the Damping Capacity Analysis (DCA). This method involves mechanically applying a certain force to the implant post in order to measure the implant's fluctuations in both the longitudinal and lateral directions [8]. The current study was done to assess the Osstell ISQ system's reliability for measuring implant stability through a cross-sectional investigation.

## Material and Methods:

The current cross-sectional study aims to evaluate the system dependability of Osstell ISQ in measuring the stability of dental implants. The research was done in the department of Prosthodontic and Oral implantology. The approval from institutional ethics committee and informed consent from all the participants was obtained prior to procedure. This prospective cross sectional clinical study was done from 2022 June to 2023 September. The inclusion criteria were; teeth to be replaced had to be extracted at least 3 months prior to implant insertion, patients without systemic illness. Total 18 participants were included for the study.18 patients, who were receiving dental restorations using the Nobel Biocare implant system with an osteoconductive-roughened titanium exterior surface, had a total of 60 consecutive implants implanted. Stability measurements were performed on these implants. The Nobel Biocare implant system was used for measurements. There were three available diameters (3.5, 4.3, and 5 mm) with a 5.5 mm platform, and three available lengths (8.5, 10, and 11.5 mm); the lengths were selected based on the availability of bone in each case. Every technique was carried out by a qualified individual.

The smart peg was manually attached to the implant fixture in order to be measured with the Osstell ISQ. Every gadget was operated in compliance with the guidelines provided by the manufacturer. The Osstell ISQ's manufacturer advises holding the gadget tip at an angle of roughly 45 degrees and in close proximity (2.0-4.0 mm) to the smart peg top without touching it. There was no contact at all between any portion of the transducer and the neighbouring teeth when using SmartPeg or a particular transducer for the 5.5-mm platform. The Osstell ISQ probe was positioned at a 90° angle to the main axis of the implant, about 2 mm from the SmartPeg. The vestibular probe was positioned in each instance.

Each implant had two SmartPeg transducers utilised for Resonance Frequency Analysis (RFA), and three measurements were completed; as a result, each implant had a total of six registers. Every evaluation was completed in order, independent of the location or register time. A trained individual used the Osstell ISQ system to complete the measurements for the RFA assessment. Measurements were completed during both surgery (post-surgery registration) and control appointments, as the latter were unrelated to the level of stability to be discovered. With no prosthesis pillar inserted, the transducer or SmartPeg was attached directly to the implant for registering. A combination of graphical and numerical methods was applied to analyse and debug the gathered data statistically. To examine concordance between sequential measurements taken by the same device on the same patients, intraclass correlation coefficients (ICC) were computed with confidence intervals set at 95%. The hypotheses of null coefficients in the sampled population were then investigated. Reliability was assessed using ICC (21) with 95% confidence intervals established. Student's t-test was used to compare the values of paired numeric variables between two related samples, while Friedman's test was used to compare the values of more than two samples. SPSS 21.0 (SPSS, Chicago, IL) software package for Microsoft Windows was used to finish the data analysis.

## Results:

Eighteen patients received a total of sixty implants, of which fifty-eight were put in the posterior maxillary region. The anterior maxillary area received the placement of the final two implants. There were 39 implants measuring 9 mm, 13 measuring 11 mm, and 8 measuring 12 mm in length. The total average scores that were achieved for the various groups were between 71.45 ISQ (SmartPegs II) and 71.44 (SmartPegs I). In the initial measurement, the average register obtained with SmartPeg I was 71.36 ISQ ± DT 6.008; in the subsequent measures, it was 71.31 ISQ ± DT 6.246 and 71.65 ISQ ± DT 6.176, respectively. However, the average register recorded with SmartPeg II was 71.02 ISQ ± DT 6.176 in its first measurement, and 71.58 ISQ ± DT 6.064 and 71.76 ISQ ± DT 6.021 in its second and third measurements, respectively. In terms of reliability analysis, Table 1 shows that the intraclass correlation coefficient (ICC) for both SmartPegs I and II was 0.96, with a 95% confidence interval of 0.95 to 0.97. This indicates an almost perfect degree of concordance. Measurement discrepancies between SmartPegs I and II were displayed in Table 2. Variations in SmartPeg I measurements were examined and categorised based on the variations: 0 indicates the same value; 1-3, 4-5, and >5 indicate differences of more than five ISQ points.

As a result, 78% of the second transducer's register and 74.3% of the first transducer's register differed by three ISQ points or less. The differences between the six completed measures were then evaluated by comparing the Smart Peg I and II measurements. Based on an analysis of variance model with repeated or intrasubject measures, the intraclass correlation coefficient was utilised to assess concordance among the difference measurements done with both SmartPegs. Both SmartPegs have repeat abilities of 0.96 and Osstell ISQ reproducibility's of 0.96.

## Discussion:

Resonance Frequency Analysis (RFA) is a non-invasive intraoral technique intended to evaluate the interface between the implant and bone, potentially offering clinical proof of implant stability. RFA makes it possible to monitor an implant through a series of stability assessments and to evaluate indirectly how osseous remodelling surrounding the implant affects secondary implant stability [3,11]. The current study used a cross-sectional analysis to evaluate the Osstell ISQ system's dependability for determining implant stability. Regarding repeatability in implant stability evaluation, we discovered that the Osstell system has very high dependability.

Using the Osstell® and AnyCheck® devices, Okuhama *et al.* discovered the association between primary and secondary implant stability. They recommend AnyCheck ® as a helpful tool for figuring out implant stability, including primary and secondary [9]. According to Lachmann *et al.* Osstell ISQ and Periotest shown adequate reliability in predicting the implant stability [12]. According to El-Sawy *et al.*'s conclusion, the Periotest (PTVs) and Osstell (ISQs) methods may both provide accurate evaluations of implant stability [13]. Using Periotest M (PTV), Anycheck, and Osstell Mentor (ISQ), Esposito *et al.* assessed the stability of the implant. They came to the conclusion that Anycheck has demonstrated its relative dependability in contrast to Periotest M and Osstell ISQ Mentor [14]. Osstell ISQ and Penguin RFA are only dependable when the implants are placed in stiff materials, according to Buyukguclu *et al.* Compared to Penguin RFA, Osstell ISQ is more dependable [15].The Anycheck device's dependability and the impact of the healing abutment diameter on the Anycheck values (implant stability test, IST) were investigated by Lee *et al.* The Periotest M and Anycheck scores as well as the ISQ and IST showed a substantial association with one another. The Anycheck readings were unaffected by the healing abutment's diameter [5]. Lee *et al.* claim that Anycheck®'s simplicity made it easier to operate and more accessible than the Osstell® Beacon+ [10]. Studies have shown that implant stability could be accurately measured using the Periotest and Osstell ISQ devices [5].The link between RFA and DCA device outcomes, which show the stability of the same implant, has been documented in a number of earlier investigations. A study conducted in vitro revealed a robust association between the outcomes obtained using RFA and DCA devices [16]. Additionally, after surgery and two months later, Pang *et al.* demonstrated a high correlation between the ISQs and PTVs [17]. Reynolds *et al.* assessed the impact of several clinical features on the values generated by the devices throughout these three intervals. They came to the conclusion that values recorded between the two measurement devices at implant placement, implant exposure, and three years after installation exhibited a weak to moderate degree of correlation [8]. The implant treatment's outcome is dependent on the implant's insertion torque (IT) in the bone. Bone resorption is encouraged when the IT is elevated. IT optimisation is thought to be essential for a successful implant therapy outcome [9]. Using the AnyCheck® device, Al-Jamal *et al.* showed a substantial association between primary stability and IT in clinical practice [18]. Using resonant frequency analysis, Haseeb *et al.* assessed the insertion torque (IT) and implant stability of two distinct implant macro geometries in various bone densities. When comparing the new implant macrogeometry to the conventional implant macro geometry, they discovered a lower IT value. No correlation was seen between IT and implant stability [19].The Osstell® is a well-established tool that has been the subject of numerous researches. However, in order to assess implant stability, it is necessary to remove the healing abutment and connect the smart peg, which may have drawbacks and inconveniences. Osstell Beacon®, which was just released, operates wirelessly. It still needs a smart peg, though, and the healing abutment needs to be put in and taken out. According to Esposito *et al.*, there was 0.16 mm of bone resorption annually as a result of the healing abutment being removed three times after implantation until the superstructure attachment period [14]. We discovered that the Osstell system is reliable for assessing implant stability. Additional research is required to validate the results.

## Conclusions:

The findings of this study suggest that, following statistical analysis using the intraclass correlation coefficient, the resonance frequency analysis system Osstell ISQ exhibits "near perfect" repeatability and reproducibility. Thus, it can be said that in terms of repeatability, Osstell system measurements are quite dependable. As a result, a single measurement can be enough.

## Figures and Tables

**Table 1:** The ISQ results' mean and standard deviation using both transducers

**Transducer**	**Measure**	**Mean ±SD**
	1st	71.36 ±6.008
Smart Peg I	2nd	71.31 ±6.246
	3rd	71.65 ±6.176
	1st	71.02 ±6.064
Smart Peg II	2nd	71.58 ±6.368
	3rd	71.76 ±6.021

**Table 2:** Measurement variations between global (all six measurements using both SmartPegs) and SmartPegs I, II

SMARTPEG I		**SMARTPEG II**		**GLOBAL (BOTH SMARTPEGS)**	
Differences	**Frequency %**	**Differences**	**Frequency %**	**Differences**	**Frequency %**
0	16	0	12	0	2
03-Jan	32	03-Jan	40	03-Jan	39
05-Apr	8	05-Apr	5	05-Apr	11
>5	4	>5	3	>5	8
Total	60	Total	60	Total	60
